# Recent advances in acute pain management: understanding the mechanisms of acute pain, the prescription of opioids, and the role of multimodal pain therapy

**DOI:** 10.12688/f1000research.12286.1

**Published:** 2017-11-29

**Authors:** Richa Wardhan, Jacques Chelly

**Affiliations:** 1 Department of Anesthesiology, College of Medicine, University of Florida, Gainesville, Florida, USA; 2 Department of Anesthesiology, Posner Pain Center, University of Pittsburgh Medical Center, UPMC Presbyterian-Shadyside Hospital, Pittsburgh, USA

**Keywords:** acute pain, pharmacogenetics, psychosocial factors, multimodal therapy

## Abstract

In this review, we discuss advances in acute pain management, including the recent report of the joint American Pain Society and American Academy of Pain Medicine task force on the classification of acute pain, the role of psychosocial factors, multimodal pain management, new non-opioid therapy, and the effect of the “opioid epidemic”. In this regard, we propose that a fundamental principle in acute pain management is identifying patients who are most at risk and providing an “opioid free anesthesia and postoperative analgesia”. This can be achieved by using a multimodal approach that includes regional anesthesia and minimizing the dose and the duration of opioid prescription. This allows prescribing medications that work through different mechanisms. We shall also look at the recent pharmacologic and treatment advances made in acute pain and regional anesthesia.

***“Nobody can go back and start a new beginning but anyone can start today and make a new ending.”***

Maria Robinson

## Introduction

The over-prescription of opioids and the exclusion of other non-opioid options have contributed to a national opioid epidemic crisis. It is estimated that the use of opioids costs over $700 billion annually. In the wake of the opioid crisis, various federal and non-federal agencies introduced stricter guidelines to address prescription practices and prevent this situation from getting worse.

A recent CDC report indicated that medical prescriptions of opioids peaked in the US in 2010 and have declined each year since, while in contrast opioid overdose deaths continued to rise. The most recent increase in the use of opioids is widely attributed to the illicit non-medical use of heroin, fentanyl, and now carfentanyl (50 times more potent than fentanyl).

The CDC report indicated that the dose and duration of the treatment represented important factors leading to addiction. In this report, it was also suggested that a treatment as short as 10 days can lead to opioid dependency. Recent data also suggested that up to 15% of surgical patients may become dependent following the perioperative use of opioids. This re-enforces the recommendation of limiting the amount of opioids used during the perioperative period and favors a multimodal approach that includes regional anesthesia.

Pain is multidimensional in nature, injury is just one cause of pain, and various factors play a role in the pathogenesis of pain. In recent years, the role of psychosocial factors in the level of pain experienced by patients undergoing surgery has been at the forefront of research.

## Remodeled pain management guidelines

In the wake of the prescription opioid abuse crisis, the American Pain Society (APS) and the American Society of Anesthesiologists (ASA) have developed a comprehensive evidence-based guideline for postoperative pain management. Of the numerous recommendations, four were considered high-quality evidence and hence strongly recommended ^1^.

1.  “…that clinicians offer multimodal analgesia, or the use of a variety of analgesic medications and techniques combined with nonpharmacological interventions, for the treatment of postoperative pain in children and adults”
2.  “…that clinicians provide adults and children with acetaminophen and/or nonsteroidal anti-inflammatory drugs (NSAIDs) as part of multimodal analgesia for management of postoperative pain in patients without contraindications”
3.  “…that clinicians offer neuraxial analgesia for major thoracic and abdominal procedures, particularly in patients at risk for cardiac complications, pulmonary complications, or prolonged ileus”
4.  “…that clinicians consider surgical site–specific peripheral regional anesthetic techniques in adults and children for procedures with evidence indicating efficacy”

There has also been a need for classifying the multidimensional aspect of pain. Recently, a task force on the taxonomy of acute pain by the APS and the American Academy of Pain Medicine (AAPM) has come together to standardize the clinical diagnostic criteria for pain ^2^.

The group proposes a five-dimensional approach to describe acute pain conditions:

1.  Core criteria: this dimension describes the inciting acute pain event and allows a given acute pain condition to be diagnosed and distinguished from other acute pain conditions
2.  Common features: emphasis is on the signs, symptoms, and qualities of each acute pain condition and includes a special stress on the temporal, spatial, and anatomical distribution and anticipated recovery
3.  Modulating factors: this element stresses the comorbid medical conditions, socio-demographical, biological, clinical, and behavioral factors associated with the pain experience
4.  Impact/functional: this constituent includes the trajectory to recovery, e.g. physical, social, psychological (pain catastrophizing), and vocational consequences resulting from acute pain conditions
5.  Putative pain pathophysiology mechanisms: this dimension highlights the relevant neurobiological pathways prior to, during, and after the initiating event

Such a complex approach clearly reflects on the multidimensional nature of acute pain, the complexity and diversity of the mechanisms involved, and the tissue’s specificity of the response to an “insult” ^2^.

## Psychosocial factors contributing to pain

A meta-analysis reviewing the literature on the role for anxiety and pain catastrophizing (defined as the tendency to magnify/dread the pain and feel helpless in the context of pain) concluded that there is good evidence that psychosocial factors play a major role in the development of chronic pain ^3^.

A very recent trial called the SCOPE trial (Stepped Care to Optimize Pain care Effectiveness) studied the independent effects of depression, anxiety, and pain catastrophizing on pain outcomes. The authors found that improvements in depression, anxiety, and pain catastrophizing predicted reductions in pain intensity and pain-specific disability. It has also been concluded that prior operative improvement in depression, catastrophizing, and anxiety has positive benefits. Accordingly, it seems that depression is the most important factor, followed by pain catastrophizing, and anxiety is last. Also, the beneficial effects of psychological improvement on pain outcomes were significant, even after adjustment for the effects of the optimized analgesic intervention ^4^.

Preoperative risk factors were thought to predict pain catastrophizing and depression in patients scheduled to have joint surgery. At-risk patients were those with high preoperative pain scores in the presence of good objective clinical function; in addition, those who subjectively rated increased pain and poor function, younger patients, females, and patients with multiple comorbidities are also at significant risk for hospital anxiety and depression ^5^.

A recent clinical practice guideline from the American College of Physicians stresses the importance of using integrative health modalities in treating patients with low back pain. Therapies given strong recommendations were mindfulness-based stress management ^6^, acupuncture, exercise, and multidisciplinary rehabilitation ^7^. The concept of multidisciplinary rehabilitation involves bringing several healthcare providers together and targeting different dimensions of recovery like pain relief, functional restoration, improvement of psychological distress, or improvement in work ability of chronic pain/opioid-dependent patients ^8^. The challenge is bringing this degree of care to the primary care level. Learning from the Swedish example, where medical clinics were offered a financial incentive for every patient who completed a rehabilitation program, might facilitate access to enhanced rehabilitation services ^8^.

## Multimodal pain therapy

Multimodal analgesia involves the concurrent use of primarily non-opioid analgesics to take advantage of the additive, if not synergistic, effects that produce superior analgesia while decreasing opioid use and opioid-related side effects ^9^. Also a key component of multimodal pain management is the utilization of regional analgesic techniques, including peripheral and field blocks and neuraxial blocks (epidural analgesia, for example).

The ASA task force on acute pain management in 2012 recommended that multimodal pain management should be included for the management of perioperative pain whenever possible. The ASA task force members recommended that acetaminophen should be considered an integral part of a postoperative multimodal pain management regimen and that cyclooxygenase (COX)-2-selective NSAIDs, non-selective NSAIDs, and calcium channel α-2-δ antagonists (gabapentin and pregabalin) should be considered as part of a postoperative multimodal pain management regimen. Moreover, guidelines suggest that patients should receive an around-the-clock regimen of NSAIDs or acetaminophen. Whenever the patient’s characteristics allow, regional blockade with local anesthetics should be considered as part of a multimodal approach for pain management ^10^. Other potential drugs to be used include steroids, α-2-agonists such as clonidine and dexmedetomidine, N-methyl-D-aspartate (NMDA) receptor antagonists, with ketamine as the primary example, but also dextromethorphan, and magnesium administered locally, intravenously (IV), and/or as a part of the local anesthetic mixture. However, it should be recognized that to date the efficacy of a given “cocktail” has not been studied. The principles behind the use of such “cocktails” are really theoretical. This is probably an area of research that deserves the most attention.

### NSAIDs

NSAIDs have been recognized to decrease opioid consumption by 25–30%, provide superior analgesia when combined with opioids, and have been proposed as first-line medications for mild-to-moderate pain, but NSAIDs are associated with side effects, namely colonic/diverticular bleeding, consistently ^11^. The efficacy and toxicity of NSAIDs result from their inhibition of COX, which exists in two forms: COX-1 and COX-2. COX-2-selective inhibitors (coxibs) display significantly lower gastrointestinal toxicity than do traditional NSAIDs. However, coxibs are associated with an increased risk for cardiovascular events.

### Acetaminophen

Acetaminophen is a non-opioid, antipyretic analgesic whose mechanism of action is still not completely understood. It is highly selective and has an additive and not necessarily synergistic effect when combined with NSAIDs. The present guideline recommends to not exceed 3 g/day in an average-sized adult to avoid liver toxicity. Acetaminophen differs from other NSAIDs, as it lacks significant anti-inflammatory activity. Recent guidelines recommend its use as a scheduled dose rather than *pro re nata* (PRN; when necessary) and in combination with NSAIDs ^9^. The other formulation of acetaminophen is the IV one. Sinatra *et al*. conducted a randomized controlled trial where IV acetaminophen was first tested in orthopedic patients ^12^. They reported that it was efficacious for moderate-to-severe pain. The use of IV acetaminophen has been evaluated in studies and various surgeries demonstrating that multimodal analgesia with this drug as a component is well tolerated and decreases opioid consumption ^12, 13^. It is particularly useful perioperatively when oral medications are not recommended. It seems that similar benefits can be achieved with the preoperative administration of *per os* (PO; by mouth) acetaminophen compared to an intraoperative IV infusion. Therefore, IV acetaminophen is likely better indicated in patients unable to take medications PO.

### Tramadol

Tramadol is a weak opioid agonist and has two mechanisms of action: binding to the μ-opioid receptor and inhibiting the reuptake of serotonin and norepinephrine. Theoretically, its weak opioid effect makes it desirable (less respiratory depression, pruritus); however, tramadol is a substrate for the cytochrome P450 CYP2D6 liver enzyme, so any agents with the ability to inhibit or induce this enzyme will probably interact with tramadol. It should be used with caution in patients on SSRIs for fear of precipitating increased release of serotonin (serotonin syndrome). Presently, there is a lack of evidence for the benefit of tramadol ^14^. However, no evidence supports the concept that tramadol is less addictive than any other opioids.

### NMDA

The NMDA receptor plays an important part because of its role in central sensitization. Central sensitization through the descending pathway involves a nociceptive signal that is potentiated in the peripheral nervous system, causing hyperexcitability in the spinal cord ^14^ that is thought to be involved in inducing chronic and/or neuropathic pain. Therefore, the dampening of central sensitization has played an important role in the prevention and treatment of both postoperative pain and chronic pain ^15^.

Ketamine, magnesium, methadone, and dexamethasone all have NMDA-blocking ability, but ketamine has emerged as a front-runner in the perioperative period. A review of available trials concluded that ketamine alone ^16^ or added to morphine/hydromorphone showed mild improvement in postoperative analgesia while reducing opioid requirements along with reducing postoperative nausea and vomiting (PONV) ^17^. An IV bolus before incision and then a continuous infusion is an effective option for postoperative pain control ^18^. If the infusion is administered over a prolonged period of time (48 hours) for more invasive and routinely painful procedures, patients can be at lower risk for developing persistent postoperative pain in the subsequent months ^15^.

Intranasal ketamine provides a safe and efficacious alternative to intranasal fentanyl with the potential added benefit of decreasing opioid use ^19^. Ketamine has a bioavailability of 45–50% when administered through the intranasal route, and a dose of 1 mg/kg provides absorbed drug levels in the subdissociative range ^15^.

### Gabanoids, gabapentin, and pregabalin

Gabanoids, gabapentin, and pregabalin are anticonvulsants but are also called neuromodulators, as they reduce neuronal excitability by inhibiting the α-2-δ subunit of calcium-gated channels on presynaptic axons. Although several single clinical trials report that gabanoids decrease the postoperative use of opioids, the Pfizer multi-centric phase III program failed to confirm these findings and to demonstrate any effectiveness in three different models ^20^. Nevertheless, the use of gabanoids for acute pain is extensive, even if it is not an approved indication. Recently, the non-approved use of gabanoids has been questioned ^21^.

Many of the gabanoid trials were uncontrolled or ineffectually controlled and of short duration ^20^. In a recent meticulously conducted placebo-controlled trial, pregabalin was found to be ineffective for patients suffering from sciatica ^20^.

### Fixed-dose combinations

Several analgesic drug combinations have been tested for the management of postoperative pain, e.g. hydrocodone/oxycodone with Tylenol, which work synergistically and help to reduce the pill burden. Combinations like this also pose risk to the body as in hepatotoxicity from acetaminophen (>3 g). Newer combinations like those combining NSAIDs and opioids have a better safety profile. Hydrocodone/ibuprofen (7.5 mg/400 mg) and oxycodone/ibuprofen (5 mg/400 mg) are two oral, fixed-dose combination formulations approved in the United States for the short-term (up to 7 days) management of acute, moderate-to-severe pain ^22^. Another novel combination of dexketoprofen and tramadol not available in the United States promises to counteract the side effects of both groups while providing additive analgesia ^22^.

### Regional anesthesia

Utilizing regional anesthesia to block peripheral or central nerves is an effective option to reduce or eliminate the need for opioids. A continuous infusion of local anesthetics should be considered in patients requiring prolonged analgesia, who are at risk of local anesthetic overdose, and in whom motor function needs to be preserved, like patients undergoing lower extremity joint replacement. Other benefits of continuous nerve block include reduction in hospital resource utilization, decreased risk of nausea and vomiting, early discharge, and improvement in patient satisfaction ^23^.

Recently, the focus has been on the use of additives including buprenorphine, steroids, dexmedetomidine, Toradol, etc. to prolong the duration of a single injection block. Alternatively, liposomal bupivacaine has also been proposed as a replacement to continuous nerve block techniques. It should be noted that both additives and liposomal bupivacaine have been shown to produce both a sensory and a motor block. In many cases, motor block might not be desirable postoperatively. The most recent additive in the literature is dexmedetomidine. It is a highly selective α2-adrenergic receptor agonist that is an effective sedative and analgesic ^24^. A recent meta-analysis investigating the effectiveness of dexmedetomidine concluded that while there was prolongation of sensory block and improvement in onset and quality of the block, those benefits should be weighed against the increased risks of motor block prolongation and transient bradycardia and hypotension ^25^. The use of field blocks including transversus abdominis plane block, quadratus lumborum block, and serratus intercostal plane block, etc. have also been increasingly advocated.

Field blocks are non-specific, and the local anesthetic mixture is administered in fascial planes between muscles requiring a larger volume in most cases of concentrated solution compared to the concentration used to perform peripheral nerve blocks for perioperative analgesia. They also require the use of ultrasound and are mostly single injections lasting only a couple of hours. There is no evidence that field blocks are as effective as peripheral nerve blocks for the same indication.

In the past few years, the use of neurotoxins such as saxitoxin, known for its blocking properties of the voltage-gated Na ^+^ channel, is being considered as an alternative to local anesthetics for regional anesthesia. Following the administration of neosaxitoxin in several animal models and a phase I study conducted in humans, it was demonstrated that neosaxitoxin alone produces minimum nerve block conduction but that the combination of neosaxitoxin and bupivacaine prolonged the duration of a nerve block ^26^. Additional studies are required to determine the role of such a toxin in regional anesthesia. Steroids like dexamethasone have been proposed for their anti-inflammatory properties. Again, there is no evidence that their local administration as part of the block produces greater effects than when administered intravenously ^27^.

## History of the opioid epidemic

In 2000, the *Joint Commission on Accreditation of Healthcare Organizations (JCAHO*) released standards for pain management, including the introduction of pain scales in recovery rooms and criteria of an acceptable pain score for discharge. The concerns regarding these new standards peaked after it led to an over-prescription of opioids in many hospitals. In response to this issue, the JCAHO, in 2001, declared that pain should be considered the fifth vital sign, a statement which would later be removed from subsequent recommendations ^28^. However, it did not help that pharmaceutical marketing initiatives notified doctors that a safe drug (OxyContin) was available to treat chronic pain ^29^ and that this drug could not be misused/abused because of its formulation. It was not until 2007 that Purdue pleaded guilty to federal criminal charges of misleading doctors when it claimed OxyContin is less likely to be abused ^29^.

Although, OxyContin is approved for only chronic pain, the drug is often prescribed during the perioperative period to “reduce” the dose and frequency of short-acting opioids. Frequently, 10–20 mg of OxyContin is ordered to be administered immediately prior to surgery and until discharge.

**Figure 1.**
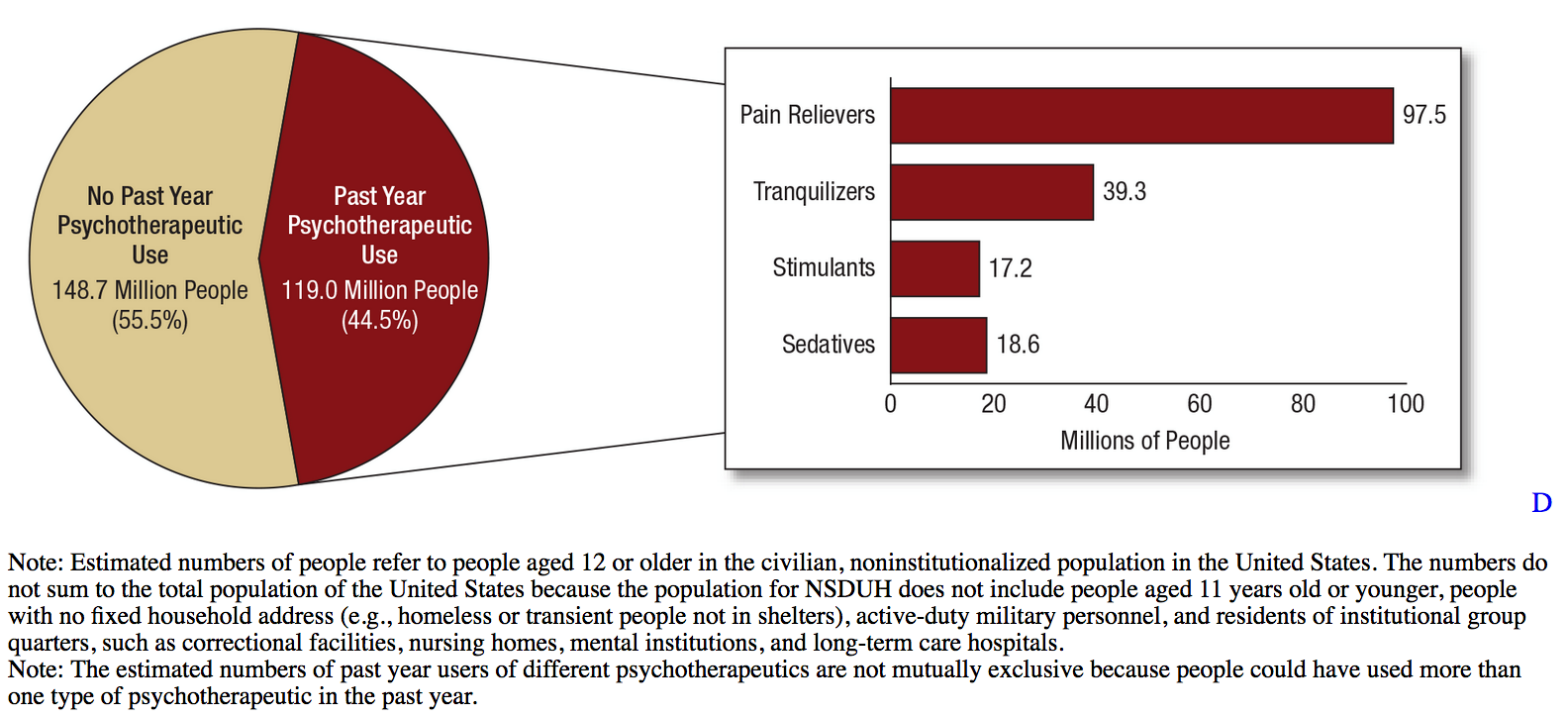
Numbers of past year prescription psychotherapeutic users among people aged 12 or older: 2015 ^31^. NSDUH, National Survey on Drug Use and Health.

**Figure 2.**
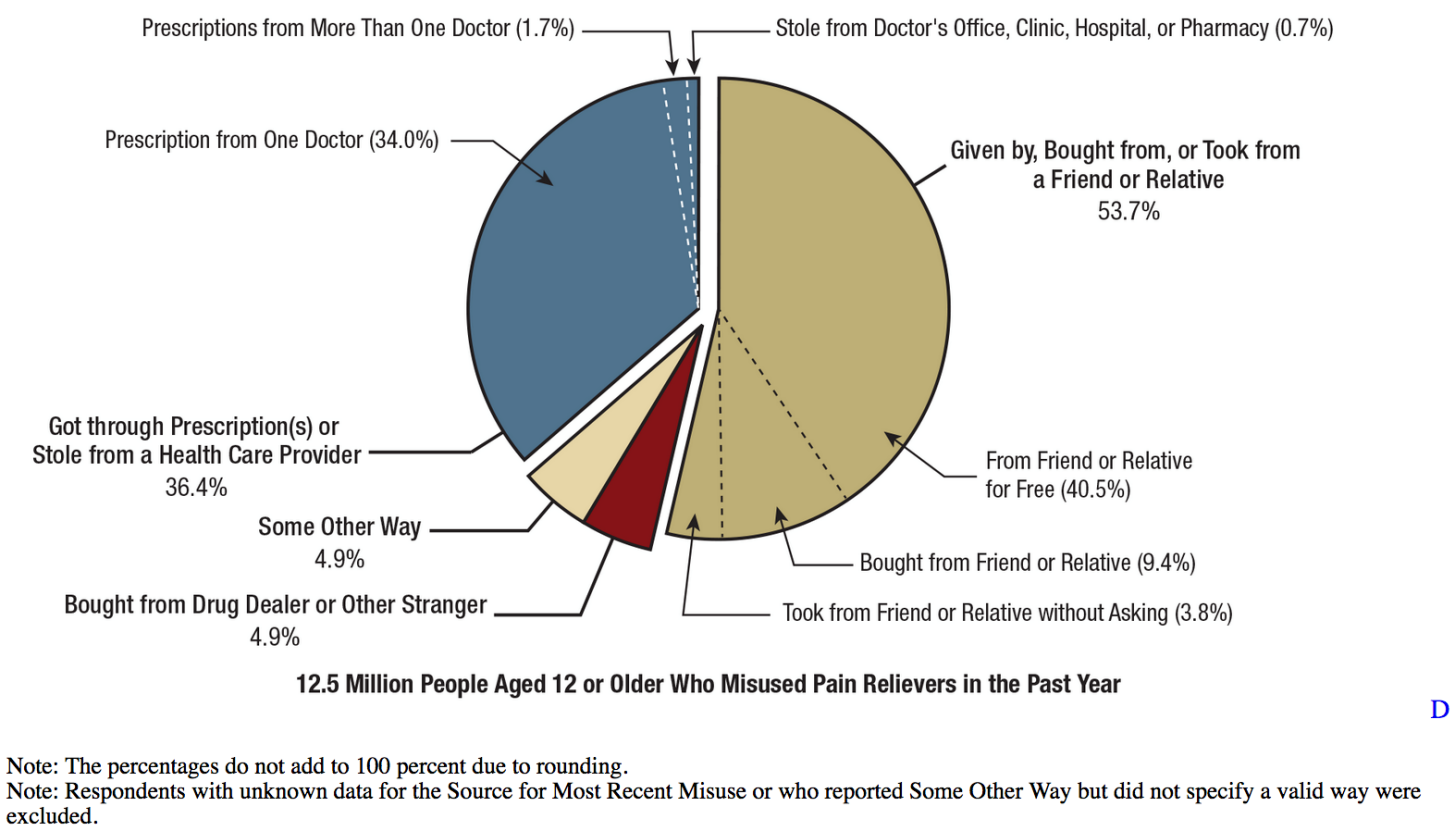
Source where pain relievers were obtained for most recent misuse among people aged 12 or older who misused prescription pain relievers in the past year: percentages, 2015.

The above factors played a significant role in the development of the opioid epidemic as we see it today. Per the 2015 National Survey on Drug use and Health, about 2.7 million people aged 12 or older had a prescription drug use disorder in the past year ( Figure 1). In 2015, 822,000 people received treatment for the misuse of pain medications ^30^. What was most disturbing is that 34% of misused prescription opioids come from legitimate physician medical practice offices ( Figure 2). Opioid overdose is now the first leading cause of accidental death in the United States, surpassed only by motor vehicle accidents ^31^.

Per a recent morbidity and mortality report released by the CDC (Centers for Disease Control and Prevention), an opioid-naïve patient showed increase risk for chronic opioid use with each additional day of medication supplied, starting with the third day, with the sharpest increases in chronic opioid use after the fifth and thirty-first day on therapy. The CDC recommends that prescribing physicians evaluate the need for a second prescription ^32^.

In surgical subpopulations, the following patients are at higher risk for opioid addiction/dependence ^33^: those who are younger, have low socioeconomic status, have preoperative pain and comorbidities (pulmonary disease, heart failure), abuse substances or tobacco, and are on specific pharmaceuticals (like benzodiazepines).

## Opioid dependence treatment programs

The four medications available for opioid dependence are methadone, buprenorphine, naloxone, and naltrexone, alone or in combination. Even though they are effective treatments, more often patients switch their addiction from one opioid to another and thus are unable to emerge from the vicious cycle of opioid dependence ^34^. The newest implant approved by the US Food and Drug Administration (May 2016) is Probuphine ^®^, which delivers steady-state levels of buprenorphine over 6 months. Numerous studies have demonstrated its efficacy and safety ^34^.

The present recommendation includes a medication-assisted treatment provided by an opioid treatment program. It combines behavioral, cognitive, and pharmaceutical therapies. Since it is clear that there is a shortage in the number of treatment centers in the country, consideration has been given to training and permitting primary care physicians to prescribe buprenorphine, thus increasing treatment accessibility ^35^.

Another barrier to be recognized is the fact that many of these patients don’t have medical insurance and therefore are directly responsible for the cost associated with it. There is no doubt that offering universal medical insurance access would allow many more patients to access opioid addiction programs readily.

There are two proposed approaches on how to manage these patients in the case of scheduled surgery: the most logical approach is to transition the patient from an opioid partial and/or complete antagonist to a full opioid agonist 2–5 days before surgery, continue the use of opioids as needed during and immediately after surgery, and transition back to their original protocol 2–5 days following surgery. The second approach simply recommends no interruption in the treatment and increasing the perioperative dosage of opioids accordingly to overcome the blockage. It is also what happens when urgent surgery is required. It should also be noted that the management of these patients is often complicated by the facts that 1) several patients take it upon themselves to stop their treatment (go “cold turkey”) and come to surgery in acute withdrawal and 2) they often don’t want to preoperatively disclose their addiction.

## Enhanced recovery after surgery pathway

In the past few years, care guidelines have been developed to take a comprehensive approach to enhance recovery after surgery (ERAS). The ERAS pathway is founded on evidence-based measures taken perioperatively to promote faster recovery after surgery. This is not only about surgery-related elements but also about the anesthesia plan, pain management, nursing, physical therapy, and nutrition ^36^. Prior to surgery, it includes physical optimization, counseling, avoidance of preoperative bowel preparation, limited fasting, and preoperative intake of carbohydrate-rich drinks. The anesthesia is characterized by the intraoperative use of short-acting anesthetics and the limited use of opioids, the perioperative period focus on the prevention of thromboembolism and antibiotic prophylaxis, and effective analgesia. Restricted fluid therapy, prevention of hypothermia, prevention of PONV, pain management based on non-opioid drugs, early enteral nutrition, stimulation of gastrointestinal motor activity, limited use of naso-gastric tubes and post-operative drains when possible, and early mobilization and removal of urinary catheters complete ERAS strategies ^37^. There is a special emphasis on the use of regional anesthetic techniques to minimize the perioperative use of opioids. Epidural and peripheral nerve and field block protocols have been developed to achieve effective perioperative pain management and prevent postoperative ileus. In this respect, local anesthetic solutions devoid of narcotics are recommended.

Several observational studies comparing pre-ERAS with post-ERAS opioid intake have noted a substantial decrease in inpatient opioid consumption with the implementation of an ERAS pathway in a variety of surgical procedures ^9^.

## Molecular mechanisms of acute pain

TRPA1 receptors are involved in the guarding deep tissue pain resulting from the incision of the fascia and muscles but not of the skin ^38^. Previously, Choi *et al*. provided evidence in support of the presence of TRPV1 receptors in the central nervous system and their involvement in neuropathic pain. It appears that an upregulation of the central nervous system TRPV1 receptors represents a key mechanism in the transition of acute to chronic pain ^39^. In this regard, Mickle *et al*. demonstrated that the upregulation of the TRPV1 receptors led to thermal and mechanical hypersensitivity modulated by parathyroid hormone-related peptide ^40^.

## Into the future of pain medication prescribing: pharmacogenetics

A variety of genes impact narcotic and non-steroidal (NSAID) drug efficacy, including the CYP family (drug metabolism) or catechol-O-methyltransferase ( *COMT*)/ *ABCB1*/ *OPRM1* (functional receptor and transport activity for analgesia versus side effects) ^41^.

CYP2D6 plays an important role in metabolizing analgesic pro-drugs to their active narcotic metabolite, such as codeine, tramadol, and dihydrocodeine. Alterations in CPY2D6 manifest phenotypically as poor, intermediate, extensive, and ultra-rapid metabolizers ^41^.

The *COMT* gene encodes the enzyme believed to moderate the transmission of pain signals via the removal of catecholamine (i.e. dopamine, epinephrine, and norepinephrine). Reduced COMT activity appears to be related to increased pain sensitivity ^42^.

P-glycoprotein transporter (P-gp) coded by the *ABCB1/MDR1* gene is an efflux transporter. It is a major determinant of the bioavailability of many opioids such as fentanyl, sufentanil, alfentanil, morphine-6-glucuronide, and morphine-3-glucuronide. P-gp is capable of limiting the entry of some opiates into the brain and pumping a variety of drugs out of the central nervous system. It is thus an important constituent of the blood–brain barrier ^43^.

The μ-opioid receptor is coded by the gene *OPRM1*. Genetic substitutions in this gene can cause patients to require higher morphine doses and systemic drug narcotic levels for effective analgesia. All of the above genes can be pharmacogenetically tested via buccal swab ^41^.

## Conclusion

In the field of acute pain, the bigger pain management challenges facing the medical community are threefold. First is reaching out to primary care doctors and surgeons (where a large portion of the narcotic prescriptions are originating) and re-educating them on appropriate pain management techniques, including the use of a multimodal approach to decrease and/or eliminate the use of opioids. In this respect, it is critical to incorporate postoperative pain management as a core competency during the training period of primary care doctors and surgeons ^33^. It is also critical to continue our efforts towards “opioid free anesthesia and postoperative analgesia” by significantly reducing or eliminating, when possible, opioid use in hospitals and clinics around the country ^44^. Second is expanding access to opioid addiction treatment programs—these programs save lives. Third is promoting research and development related to the treatment of opioid addiction via the development of new formulations such as Probuphine and Xtampza ^®^ ER (oxycodone) based on the use of higher concentrations of the active ingredient mixed with abuse-deterrent elements, which prevents diversion of the drug to a liquid form for IV injection. It is also important to continue the search for the “Holy Grail”, which in the case of opioids is a “non-addictive opioid”.
